# Ovariotomy and Abdominal Surgery

**Published:** 1899-09

**Authors:** 


**Ovariotomy and Abdominal Surgery.** By Harrison Cripps. Pp. 624. London: J. & A. Churchill. 1898.

The main part of this book runs to 422 pages, the rest being taken up with an appendix of selected cases which are out of place and in no way enhance the value of the work. We hope that in any future edition this space will be taken up by a greater elaboration of the details described in the earlier part of the volume; for the fault of the book as a whole lies in the insufficiency of description devoted to the various subjects. The author tells us that his chief object is to record such methods as he has found valuable in his own experience; but the addition of a chapter of "Anatomy of the Abdomen" (which is beautifully illustrated by coloured plates), mainly from the pen of Mr. H. J. Waring, and others on the "Surgery of the Kidney' and the "Radical Cure of Hernia," by Mr. Bruce Clarke and Mr. C. B. Lockwood respectively, indicate that there was also a desire to make the book complete and of general use. In this, however, we do not think the author has fully succeeded, owing to the want of detail. The only chapters which have received sufficiently full treatment are those on "Ovariotomy" and "Inguinal Colotomy." Our objection is rather with what has been left unsaid than with what has been actually written, and the incompleteness of the work renders it of comparatively little value to those seeking information on abdominal surgery. This could be altered, without increasing the size of the book, in the way already suggested.

